# The London Medical Student and Other Comicalities

**Published:** 1885-07

**Authors:** 


**The London Medical Student and other Comicalities.** Selected and compiled by Hugo Erickson, M. D. Published by Dr. H. Erickson, 11 Farmer street, Detroit, Mich. Price, $2.00.

The first part of the book paints the picture of medical student life in England's great metropolis, London. It is, as could be expected from a story derived from the *London Punch*, crisp and readable throughout, and exquisitely humorous. The last part of the work consists of a collection of medical anecdotes, all carefully selected and interesting. The object of this compilation is to amuse and entertain the busy doctor in his leisure hours, and those who read it will admit that its object has been attained.

